# Implementation of clinical guidelines for osteoarthritis together (IMPACT): protocol for a participatory health research approach to implementing high value care

**DOI:** 10.1186/s12891-022-05599-w

**Published:** 2022-07-05

**Authors:** Clodagh M. Toomey, Norelee Kennedy, Anne MacFarlane, Liam Glynn, John Forbes, Soren T. Skou, Ewa M. Roos

**Affiliations:** 1 School of Allied Health, Faculty of Education and Health Sciences, University of Limerick, Limerick, Ireland; 2 Health Research Institute, University of Limerick, Limerick, Ireland; 3 Public and Patient Involvement Research Unit, University of Limerick, Limerick, Ireland; 4 School of Medicine, University of Limerick, Limerick, Ireland; 5 HRB Primary Care Clinical Trials Network, Galway, Ireland; 6 Research Unit for Musculoskeletal Function and Physiotherapy, Department of Sports Science and Clinical Biomechanics, University of Southern Denmark, Odense, Denmark; 7 The Research Unit PROgrez, Department of Physiotherapy and Occupational Therapy, Naestved-Slagelse-Ringsted Hospitals, Slagelse, Region Zealand Denmark

**Keywords:** Osteoarthritis, Protocol, Implementation strategies, Guidelines, Participatory health research

## Abstract

**Background:**

The evidence-based interventions of exercise and education have been strongly recommended as part of prominent clinical guidelines for hip and knee osteoarthritis (OA) for more than ten years. Despite the wealth of strong evidence that exists, implementation in practice is sub-optimal. This paper describes the key methodologies used in the co-design, tailoring, and evaluation of the IMPACT project implementation strategies, to confront this problem across multiple levels (micro, meso, macro) in public and private healthcare settings in Ireland.

**Methods:**

Using a type III hybrid implementation-effectiveness design, a participatory, dynamic and iterative process will be used to tailor and evaluate multi-level implementation strategies using the following stages: 1) Co-design the implementation strategies with key stakeholders using best evidence, a theory-driven implementation framework (Consolidated Framework for Implementation Research), local context and expert consensus; 2) Pilot and evaluate the implementation strategies by training physiotherapists to deliver the evidence-based Good Life with osteoArthritis Denmark (GLA:D®) education and exercise programme using the implementation strategies, and conduct a mixed-methods process evaluation; 3) Adapt the implementation strategies based on implementation process evaluation indicators from stage two. The adapted strategies will be used for scale-up and sustainability in subsequent GLA:D® Ireland training programmes that will be rolled out nationally. Evaluation of effectiveness on patient and cost outcomes will continue up to 12 months post-programme delivery, using an online patient registry and pre-post design.

**Discussion:**

This implementation science project aims to use participatory health research to address a gap in management of OA across public and private healthcare settings. This research has the potential to change practice and promote a policy of exercise and physical activity referral for chronic musculoskeletal disease that utilises community engagement effectively and enacts change ‘together’, with involvement of researchers, decision-makers, clinicians and patients.

**Supplementary Information:**

The online version contains supplementary material available at 10.1186/s12891-022-05599-w.

## Background

Osteoarthritis (OA) is a leading cause of disability and one of the fastest growing health problems in the world. From 1990 to 2019, the prevalence has risen by 48% globally to 528 million people worldwide [1] and this increase is expected to continue due to population ageing and obesity epidemic amongst other factors [2]. Irish data from The Irish Longitudinal Study on Ageing (TILDA) suggests that patients with OA make significant more use of general practitioner (GP) and outpatient services than those without OA, at an annual cost of €13.6 million [3]. The highest proportion of direct costs of the disease are attributed to orthopaedic surgery and in-patient hospital stays, with smaller proportions accounted for by medications, physician visits, other health professional visits and diagnostic procedures [4]. Substantial indirect costs include productivity losses from absenteeism, presenteeism (disease-related loss in productivity that occurs even when the person is at work), premature death and early retirement (increased social welfare costs and reduced tax revenue) [4]. In Ireland, a two-tier public-private healthcare system (similar to that in Australia and, to a lesser extent, the UK) often results in inequities in access to care, and a greater burden and waiting time for those less able to pay for access to private health services. This is exemplified by the results of the Euro Health Consumer Index 2018, which saw Ireland place last of 35 countries, in terms of healthcare accessibility [5]. Access to public healthcare comes through GP referral, with 60% of the population (those without medical card or insurance) paying out of pocket on average €52 per GP visit and 75% of the population paying up to €144 per month for drugs as well as paying for other primary care services [6], creating an additional burden on those with long-term chronic disease.

Despite being a typical chronic disease characterised by long duration, current management practices for OA are best described as reactive and palliative [4]. Too much focus is on end-stage joint replacement surgery and pain-relieving procedures or medications in the interim, at a significant cost to society and the individual. There is increased recognition across healthcare organisations and the field of musculoskeletal pain, that the overuse of procedures, testing, and medications constitutes low-value care which strains the healthcare system and causes unnecessary stress and harm for patients [7, 8]. Ireland has substantial orthopaedic waiting list times that have recently been exacerbated by COVID-19 pandemic-related cancellations. As of July 2021, over 84,000 patients across Ireland were waiting for an orthopaedic outpatient appointment, with 45% of these waiting over 1 year. Yet, even in the absence of a clear complication, total joint replacement does not always provide relief: up to 30% of total knee replacement patients remain dissatisfied with their surgical result after 1 year [9]. With the projected growth and cost of this disease, the sustainability of this health-care model is questioned. OA should be viewed as a chronic condition, where prevention and early comprehensive care models are the accepted norm, as is the case with other chronic diseases e.g. cardiovascular disease. A paradigm shift from tertiary to secondary prevention strategies in the form of early exercise intervention and education is required, in order to limit progression and minimise the health consequences of the disease.

Despite a wealth of strong evidence from over 60 randomised controlled trials (RCTs) [10, 11] and clinical guidelines [12, 13] supporting the efficacy of secondary prevention strategies such as exercise and education as first line treatment strategies for patients with painful knee and hip OA, implementation in clinical practice is suboptimal. An international meta-analysis on community care demonstrated that only 35–39% of patients with hip or knee OA received appropriate exercise recommendations or education/self-management options according to the guidelines [14]. Previous international programmes such as the Good Life with osteoArthritis from Denmark (GLA:D®) initiative have aimed to address this gap in care. This initiative is designed to support the implementation of guidelines for the treatment of hip and knee OA in clinical practice [15]. The programme consists of three main components: two to three sessions of evidence-based patient education, a six-week, twice-per-week physiotherapist-supervised neuromuscular exercise training programme, and patient registry to collect outcomes. Data from 9825 patients in the GLA:D® registry demonstrated improved pain intensity and quality of life at 3 months and 12 months after starting the programme [15]. Furthermore, physical function and physical activity improved, fewer patients took painkillers following the treatment (− 24%), and fewer patients were on sick leave due to their OA joint (− 44%) at 12 months compared with the year prior [15]. In addition, the programme has shown cost-effectiveness in primary care in Denmark, with healthcare costs per Quality-Adjusted Life Year (QALY) below conventional thresholds for willingness-to-pay [16]. Cross-cultural adaptation has proven feasibility, with results replicated across other countries such as Canada and Australia [17, 18].

Research highlights that the successful implementation of evidence into practice requires a comprehensive approach adapted to the translation of evidence into a specific setting with local stakeholders so that barriers to implementation in the local context can be understood and addressed [19, 20]. While previous research has highlighted the large number of barriers to implementation of OA interventions [21–23], the main challenge in this area today is to design and tailor actionable strategies that address these barriers. Implementation strategies are methods or techniques used to improve adoption, implementation, sustainment, and scale-up of interventions [24]. Strategies vary in their complexity and can be multi-faceted and influence multiple barrier levels. In the literature, limitations of implementation strategy development include inadequate identification of the contextual determinants (i.e. barriers and enablers) of implementation, little use of theory in designing or tailoring strategies and poor reporting of monitoring and evaluating strategies [25–27]. Applying the paradigm of participatory health research to implementation strategies has the potential to improve the uptake, adherence and sustainability of healthcare interventions. The goal of participatory health research is to maximise the participation of end-users in all stages of the research process. Research is not done “on” people as passive subjects providing “data” but “with” them to provide relevant information for improving their lives [28]. Some benefits of the participatory health research approach include explicit attention to culturally and logistically appropriate research, enhanced recruitment capacity, generation of professional capacity and competence in stakeholder groups, productive conflicts followed by useful negotiation, an increase the quality of outputs and outcomes, an increase in the sustainability of project goals beyond funded time frames and greater likelihood of system and policy changes [28]. This approach recognises the value of diverse stakeholders’ (patient/community members, clinicians, academics, health planners) contributions to the co-creation of knowledge in a process that is not only practical, but also collaborative and empowering [29]. It has been used successfully in Irish primary care implementation research before [30].

This protocol will describe the key methodologies used in the IMPACT project. IMPACT aims to use a participatory health research approach to co-design, tailor and evaluate implementation strategies to optimally implement an evidence-based exercise and education programme for hip and knee osteoarthritis in the Irish health setting.

## Methods

### Design, participatory approach and theoretical framework

A plain English language summary of this research can be found in Additional file 1. This research is defined as a hybrid effectiveness-implementation trial design (type III), which tests the ability of an implementation strategy to enhance use of an evidence-based intervention while collecting data on the health impact of the intervention during implementation [31]. Using a participatory health research approach, key stakeholders are supporting the co-design and evaluation of implementation strategies that will aim to embed an evidence-based programme for hip and knee osteoarthritis (GLA:D®) in the Irish healthcare system, across public and private settings. For the purpose of this project, a stakeholder is defined as anyone who is affected by or is involved in the development of and/or delivery of the research programme [28]. The four key criteria for meaningful involvement of stakeholders in participatory health research include identifying the research questions, sharing governance and decision-making, collaborative data interpretation and shared dissemination [28]. Accordingly, this research requires stakeholder input at each of these levels during each work package to ensure successful outcomes (described in detail below).

It is valuable to combine participatory health research with implementation theory because they offer a combined heuristic device to understand and support implementation processes [32]. The study is guided by the Consolidated Framework for Implementation Research (CFIR). The CFIR is a conceptual framework developed by Damschroder and colleagues to guide systematic assessment of multilevel implementation contexts (micro-meso and macro levels) and aid identification of appropriate expert-defined domains and constructs (individual characteristics, intervention characteristics, inner setting, outer setting, process) [33]. Used prospectively, the identification of these theoretical constructs can assist the researchers and contributors to pre-empt, or identify, factors that will positively or negatively affect the implementation of best evidence [34].

The evidence-based intervention that was selected for implementation of clinical guidelines for OA was the aforementioned GLA:D® initiative. This physiotherapist-supervised group education and exercise programme for hip and knee OA was chosen for the Irish context due to existing evidence of effectiveness [15, 18], the requirement of a patient registry to monitor outcomes and the success in adapting to other international and cultural contexts and healthcare settings (public and private) [17].

The SPIRIT 2013 checklist (defining standard protocol items for clinical trials) for this study is included in Additional file 2 and the Guidance for Reporting Involvement of Patients and the Public (GRIPP-2) checklist in included in Additional file 3. Ethical approval for this study has been granted by Galway Clinical Research Ethics Committee (REC) (C.A. 2685) and University of Limerick EHS REC (2020-12-13-EHS). For scale-up of the GLA:D® initiative in additional healthcare settings, distinct ethics permissions will be sought for each region where the programme is implemented.

**Fig. 1:**
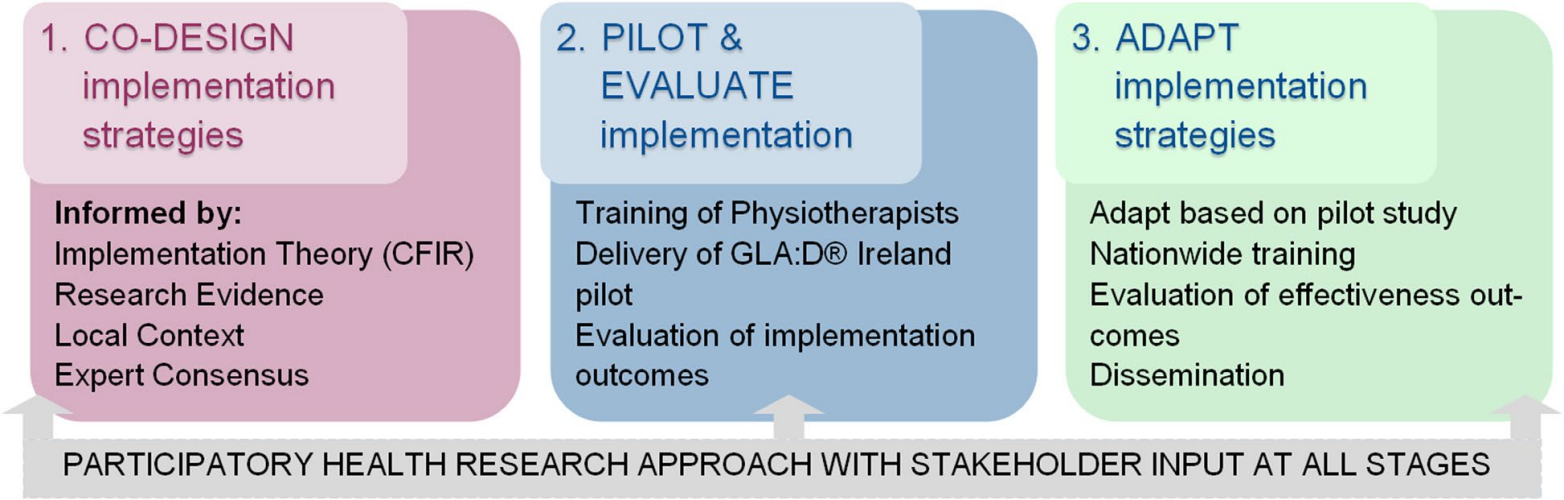
IMPACT project stages

To effectively implement clinical guidelines for OA in the Irish healthcare setting, the research methodology will involve three key inter-linked stages (Fig. 1):

1. Co-design of implementation strategies,
2. Pilot and evaluate the implementation strategies, and
3. Adapt the implementation strategies for subsequent national rollout of GLA:D® Ireland.

### STAGE 1: co-design of implementation strategies

#### Engagement of the IMPACT steering committee

A steering committee of key stakeholders with relevant research, clinical/system expertise or lived experience (researchers, patients, patient advocacy group members, physiotherapists, GPs, orthopaedic surgeon) will be recruited via word of mouth and personal communication. The main purpose of the committee is to discuss and agree on implementation strategy designs that are considered suitable to integrate GLA:D® in the existing routines of the Irish healthcare system. Three to four online meetings will take place each year to achieve this objective, along with other tasks as outlined below.

#### Co-design

The co-design and tailoring of multi-level implementation strategies will involve a series of review stages and consultations with the steering committee, using a three-step iterative process:

- STEP 1: Identify barriers and enablers to implementation informed by (i) implementation theory, (ii) research evidence, (iii) local context, and (iv) expert consensus.
  (i) Implementation Theory

The steering committee members will vote on barriers that are deemed relevant to the project using a list of theory-driven CFIR constructs (*n* = 39) by each domain [33]. These barriers will subsequently be used with the CFIR- Expert Recommendations for Implementing Change (ERIC) Barrier Busting Query Tool [35] to match with an appropriate enabler or strategy. This tool was developed by Powell et al. using a Delphi process to generate expert consensus on commonly used or effective strategies [35] and will form the initial list of barriers and enablers to implementation. To further operationalise the CFIR domain “characteristics of individuals”, this domain will be split into two, namely: “characteristics of physiotherapists” and “characteristics of patients” to address the different barriers and strategies that will be applied to each.

(ii) Research Evidence

Previous published reviews on barriers and enablers to implementation of exercise and education for osteoarthritis will be reviewed and mapped to CFIR constructs [21–23, 36, 37]. A scoping review of strategies used for the implementation of exercise programmes for chronic conditions in healthcare and community settings will be carried out. The purpose of this review is to understand the type and evaluation of existing implementation strategies used globally. Identified elements from this review will be mapped to CFIR construct headings where relevant.

(iii) Local Context

Due to the disparity between recommended exercise guidelines for OA and patient referral or access to appropriate exercise programmes, there is a need to understand the local beliefs, barriers and enablers to inform development of the implementation strategies. This will be carried out via three cross-sectional online self-administered surveys for physiotherapists, GPs and patients with OA in Ireland respectively. Three tailored online cross-sectional surveys (designed using Qualtrics®), adapted from the work of Cottrell et al. [38], Holden et al. [39] and Davis et al. [40], will be reviewed for face validity by members of the steering committee prior to distribution. All surveys will be advertised via social media (Twitter, LinkedIn) and word of mouth. In addition, the GP survey will be distributed to the Irish College of General Practitioners network, the physiotherapist survey will be distributed to the Irish Society of Chartered Physiotherapists network and the survey for people with OA will be advertised in various newsletters, community noticeboards and Arthritis Ireland social media. Exercise beliefs statements will be rated on a 5-point Likert scale from strongly agree to strongly disagree and collapsed to a binary scale (agree vs. disagree/neither) and multiple choice and open questions will be given for identification of barriers and enablers. Results of each survey will be analysed with assistance from the online CFIR codebook template to identify barriers and enablers under appropriate construct headings.

(iv) Expert/Steering Committee Consensus

Other barriers, enablers and strategies identified by steering committee members or in consultation with other relevant clinical, patient and policy stakeholder groups (e.g. Irish College of General Practitioners, National Clinical Programme for Trauma and Orthopaedic Surgery (orthopaedic consultants), Arthritis Ireland (patient and carer advocacy group), Irish Society for Chartered Physiotherapists etc.) will be sought, discussed and considered as relevant.

- STEP 2: Combine findings from (i) to (iv) in STEP 1 under each CFIR construct, to create or tailor distinct and actionable implementation strategies. The ERIC Barrier Busting Query Tool will be applied again to query any newly identified barriers. Strategies will be grouped according to targets as appropriate (e.g. patient engagement, healthcare professional engagement and training, changing policy, community partnerships). Each strategy will be mapped to the relevant CFIR construct barrier(s) and will include a method to evaluate the impact of each strategy. Reporting guidelines for implementation strategies as recommended by Proctor et al. will be used where appropriate [24]. An example is provided in Table 1.
- STEP 3: The steering committee will review each strategy and discuss relevant changes or additions. Information presented in the GLA:D® education materials will also be adapted to suit the Irish context and health system for items related to e.g. terminology, models of care and medications as relevant in this step.

**Table 1:** Example of reporting methods for implementation strategies using prerequisites suggested by Proctor et al. [24]

Implementing GLA:D in clinical practice (Name it)	
Strategy (Define it)	Specify it
1. Each PT will need to work with the clinic manager and colleagues to have dedicated time to implement. A GLA:D Ireland PT Toolkit to assist with these conversations will detail: i. Programme evidence, ii. Programme requirements in terms of time, space, equipment and process mapping, iii. Alignment with government or international policy and waiting list support, iv. Opportunities to be involved in research or improve service with data or benchmark patient or clinic outcomes.	**Actor:** Researchers, lead implementer and clinic managers/colleagues
	**Action:** Researchers produce toolkit and encourage discussion. Lead implementers initiate discussion in clinic and present case.
	**Target:** Meso-level. Number of sites trained and implementing the programme. Nature of feedback from implementers in qualitative interviews.
	**Temporality:** Toolkit presented at training course. Discussion initiated immediately following training. Re-visited as needed.
	**Dose**: Once at initiation of implementation. Re-visited as needed.
	**Implementation outcome affected:** acceptability, adoption, appropriateness
	**Justification (CFIR barrier addressed):** Availability of resources (inner setting), Relative advantage (intervention characteristics) **(References in literature):** Briggs et al. 2019, Lau et al. 2016

### STAGE 2: pilot and evaluate the implementation strategies

This stage will involve piloting the implementation strategies co-designed in stage one and evaluating the effectiveness of the strategies.

#### Training of physiotherapists

An initial convenience sample of engaged physiotherapists will be contacted via word of mouth through co-investigators and steering committee members to take part in the first GLA:D® training course and pilot study. This sample will be representative of different regions and across private (small and large clinic) and public (primary care and orthopaedic hospital) healthcare settings in Ireland. These physiotherapists will participate in a 2-day continuous professional development course giving them the requisite skills to diagnose OA and deliver care as described in the clinical guidelines. The course theory summaries latest evidence on treatment of OA, as well as practical instructions in the specific protocol of GLA:D®, including delivering patient education, supervising and instructing neuromuscular exercise and the use of the online registry [15]. The course will initially be delivered by representatives from GLA:D® Denmark, with additional training on relevant implementation strategies identified in stage one provided by CMT. A patient representative will also be present at the training to provide appropriate input. This will give the physiotherapists access to an implementation ‘toolkit’ and online platform with all the material needed to deliver GLA:D® locally in the clinic.

#### GLA:D® pilot programme delivery

GLA:D-trained physiotherapists (*n* = 8–10) will make use of existing or new referral sources to invite people with hip or knee OA to take part in the GLA:D® programme, as applicable for each setting. These sources include but are not restricted to clinic or orthopaedic waitlist, GP or other healthcare professional referral and self-referral. Eligible participants will have ‘joint problems from knee and/or hip that have resulted in contact with the health care system’. Exclusion criteria include non-OA cause of symptoms, e.g., tumour; inflammatory joint disease, or sequelae after hip fracture; other symptoms that are more pronounced than the OA problems, e.g., chronic, generalised pain, or fibromyalgia, acute knee trauma in the past 6 months or inability to understand the English language. The GLA:D® programme will comprise two to three education sessions and 12 (twice weekly) group neuromuscular exercise sessions as described in detail previously [15]. All group sessions will be supervised by a physiotherapist and can be delivered in a face-to-face or online format, as per clinician/patient preference and capability. Participants are encouraged to continue being physically active and to exercise, either with their physiotherapist or in their local community, to sustain the effects from the treatment in the long term.

#### Evaluation of GLA:D® implementation

As established by Proctor and colleagues [41], a core set of implementation outcomes including: acceptability, adoption, appropriateness, cost, feasibility, fidelity, penetration and sustainability will be evaluated as outlined in Table 2*.* Implementation outcomes will be analysed descriptively using frequencies/proportions for categorical variables and means and standard deviations or medians and interquartile ranges for continuous variables.

**Table 2:** Implementation outcomes and proposed method of collection

Outcome	Definition	Method of Collection (Level of analysis)
Acceptability	Satisfaction with various aspects of the programme	Survey on beliefs (Providers and patients); Survey on satisfaction at 3 months and 12 months post-programme (Providers and patients) Interview with providers: “Were you satisfied with the programme?” Interview with patients: “Were you satisfied with the programme?”
Adoption	Uptake and utilisation of the programme	Registry: Recording the number of referral sources, sites trained vs. sites implementing, trained physiotherapists and participant registrations from private and primary care settings (Providers and patients)
Appropriateness	Perceived fit, relevance, or compatibility of the programme for a given setting, provider, or consumer; and/or to address a particular issue or problem	Survey on beliefs (Providers and patients); Survey on satisfaction at 3 months post-programme (Providers and patients) Interview with providers: “How compatible is the GLA:D programme in your setting?” Interview with providers and patients: “Does the programme address a particular issue or problem?”
Cost	The financial impact of the implementation	Survey on barriers from WP1 (Providers and patients), Health utilisation and sick leave/medication use survey at 3 months and 12 months post-programme (Patients) Cost-effectiveness analysis Interview with providers and patients: “Have there been any cost implications?”
Feasibility	The extent to which the programme can be successfully used or carried out within a given setting	Collected with a survey at training course with strategies to overcome specific feasibility barriers discussed (Providers)
Fidelity	The degree to which the programme is implemented as it was prescribed in the original protocol	Interview with providers: “Did you make specific changes to the original programme? What did you change?”
Penetration	The integration of a practice within a service setting and its subsystems	Registry: recording the number screened during the study period and reporting of the absolute number and proportion of those who participated in the program as well as the number of referral sources (Providers);
Sustainability	The extent to which the implemented programme is maintained	Number of sites still running the programme at 12 months and by the end of the project (Providers); Physical activity and exercise participation at 3 and 12 months (Patients) Interview with providers: “Do you intend to continue delivering the programme?” Interview with patients: “Do you intend to continue doing the exercises?”

Semi-structured interviews will be conducted with 8–10 physiotherapists across healthcare settings (primary care, orthopaedic hospital, private practice) that have implemented the pilot programme within a five-month time-frame and those who have not implemented the programme, if appropriate. Semi-structured interviews will also be conducted with 12–15 patient participants across each setting. For people with OA, criterion purposive sampling will be used, seeking a representative sample based on sex, affected joint, disability level, duration of symptoms and number of attended sessions. The purpose of these interviews is to capture barriers and enablers to GLA:D® programme delivery and provide context related to implementation outcomes. The interview guide was adapted from Davis et al.’s [17] evaluation of GLA:D® in Canada and is presented in Additional file 4. Included topics relate to experiences providing or taking part in the programme and format (online or face-to face, if applicable), barriers or enablers encountered and any prior information or support that would have helped in delivering, taking part or adhering to the programme. Interviews will be recorded and transcribed verbatim. Interview data will be analysed thematically using a deductive approach primarily, with assistance from the CFIR codebook template for qualitative data [42, 43]. After multiple readings by the research team, codes will be assigned from units of the text and grouped into pre-defined themes using NVivo software (QSR International). Any additional themes identified using an inductive approached will be labelled as appropriate. Using reflexivity, the interviewer will also document pre-existing ideas and perspectives in an attempt to identify any biases [44]. A summary of findings from the interviews and identified themes will be presented to the steering committee to allow for additional interpretation and discussion.

### STAGE 3: adapt the implementation strategies

This stage will involve the review of and reflection on each implementation strategy, according to the defined method of evaluation as identified in stage one and the quantitative and qualitative pilot programme results from stage two. Reflecting and evaluating also lends itself to the ‘process’ construct under the CFIR, with a specific focus on implementation efforts, accompanied with personal and team debriefing about progress and experience [33]. Thus, the steering committee will decide on appropriate and realistic adaptations to be made to the implementation strategies based on the pilot findings. These strategies will then be used to launch the national GLA:D® Ireland programme for hip and knee OA and incorporated into future training workshops for physiotherapists. Strategies will be re-evaluated during the review of GLA:D® Ireland implementation annually and adapted where necessary.

#### Evaluation of effectiveness

An electronic GLA:D® Ireland registry will collect participant consent and all therapist- and patient-reported data from baseline, 3- and 12-months follow-up. The secure REDCap™ software platform will be used to build, collect and manage all data related to the registry and research project. An evaluation of programme effectiveness relating to self-reported and measured outcomes such as pain, joint symptoms, quality of life and functional capacity (outcomes collected presented in Additional file 5) will be performed at each annual review. Effectiveness outcomes, using a pre-post design, will be analysed to evaluate change scores (baseline to 3- or 12-month follow-up) with 95% confidence intervals to determine significance (α = 0.05) and clinical significance where a published value exists. If appropriate, a mixed-effects model will be used with patient as a random effect and time as a fixed effect. Sensitivity analyses will be conducted to determine differences in results related to site of pain (knee/hip), history of joint replacement and private or public setting. IBM SPSS® Statistics 26 will be used to perform all statistical analyses.

#### Evaluation of cost-effectiveness

A cost utility analysis will be conducted after 2 years of implementation to determine the financial impact of implementing the programme. The direct and indirect costs of implementing GLA:D® will be compared to its benefits. Using the 5-dimension EuroQol (EQ-5D) to measure health related QALYs, the raw and adjusted incremental cost-effectiveness ratio (ICER) will be calculated 1 year after the intervention. An exploration of healthcare utilisation pre- and post- participation in the GLA:D® programme will be conducted at each time-point (Additional file 3) and compared to national data from the TILDA study [45].

## Discussion

With the IMPACT project, implementation of an evidence-based initiative for OA management in Ireland will be the result of comprehensive implementation strategy development using theory, best evidence, knowledge of the local context and needs of patients and clinicians and expert stakeholder consensus. This method will be used to systematically identify the multi-level barriers that exist and co-design solutions and strategies to overcome them.

Incorporating a participatory research approach and including end-users in the planning, design, implementation, and evaluation stages is seen as a critical approach to overcoming pragmatic barriers and ensuring the appropriate care is delivered to the patients who need it. Knock-on benefits of this research approach may come from seeding engaged networks of stakeholders that advocate for change in orthopaedic and musculoskeletal practice that result in benefits to the individual, healthcare system and society. While it was not deemed feasible in the planning stages to involve a policy-maker in all steering committee activities, opportunities to engage decision-makers and funders will be sought in a timely and strategic manner. Given OA is one of the most prevalent and disabling of chronic non-communicable disease, even small improvements in care that improve patient outcomes will incur savings. A budget impact analysis in Australia stated that only one in twelve GLA:D® recipients would need to avoid knee replacement surgery for the programme to generate savings [46]. In Demark, GLA:D® has shown cost-effectiveness at 1 year [16]. It is anticipated that similar future findings in Ireland will be leveraged to advocate for improved funding and access to physiotherapy-supervised exercise and education programmes for all patients with OA, in line with clinical guidelines. This aligns well with visioned plans to achieve universal healthcare in Ireland (Sláintecare) by 2028 [6], and to ensure all patients get access to the right care, in the right place, at the right time.

Implementation strategies are further operationalised by level i.e. micro, meso, macro. It is acknowledged that certain barriers may be non-modifiable within the scope and lifetime of this research study, particularly at a macro level. Nonetheless, strategies related to policy and engagement of stakeholders will work towards tailoring messages and driving change at these levels where appropriate. It is envisaged that patient outcomes and cost effectiveness data collected after 1 year of implementation will be used to leverage and advocate to Department of Health, the Health Service Executive and other relevant bodies to fund supervised exercise and education interventions for chronic musculoskeletal conditions. It will be important to apply this data alongside international evidence to help improve models of care and pathways for people with chronic hip and knee pain.

While local context is important to include with implementation efforts, this research has relevance beyond the specific setting in which it is being conducted. As with all intervention research, implementation strategies need to be fully and precisely described, in detail sufficient to enable measurement and ‘reproducibility’ of their components [24]. The details of the participatory health research process will be reported so that implementation projects work in other care settings, for other chronic conditions and other countries can be informed by lessons learned during that process. The use of theory in this study will also enhance the scope to accumulate knowledge about overcoming barriers to implementation [34]. Therefore, the strategies deemed to be effective will be published and shared widely for potential transfer and implementation in other healthcare settings, contexts and conditions.

Dissemination activities arising from the results of this research will be co-planned and reviewed by the steering committee throughout the project. Annual reports, summarising evaluations on the effectiveness of the programme will be produced with accessible, lay terminology and infographics, and shared with clinicians and participants of the programme. Ensuring open access, these reports will also be available for free download on the GLA:D® Ireland website [47]. Sustainment of the GLA:D® Ireland initiative beyond the duration of project funding is one of the primary implementation indicators and will be addressed and tailored in implementation strategy design. The very nature of the implementation-effectiveness research design in this project will work to establish an evidence-based intervention within community healthcare settings. Nonetheless, it is critical that efforts to embed and sustain this initiative long-term are applied, through identification of the supports and processes required at micro, macro and meso levels. There is a clear need to identify and describe factors that affect sustainment outcomes in the field of implementation science, and a lack of evidence supporting discrete sustainment strategies for evidence-based interventions [48].

This research is limited by the pre-post design used to evaluate effectiveness of the intervention, without a comparative group. Since there are 100+ conclusive trials already completed in the area of exercise effectiveness for OA, the pre-post design was considered sufficient for the pragmatic monitoring of health outcomes in practice, alongside primary outcomes of implementation. This research may be subject to bias arising from the specific backgrounds and experiences of the researcher and steering committee members it is acknowledged, for example, that different group members may have prioritised or interpreted strategies and results in a different manner. Results of this research are also limited by the lack of available health administrative data to track healthcare usage in Ireland. However, using self-report, we can track use across private and public health services in this manner, that may not be feasible using health administrative data alone. This is important data to capture within a complex, two-tier system of care.

The IMPACT project will use implementation science and participatory health research to address a gap in evidence-based management of OA across public and private healthcare settings in Ireland. This research has the potential to change practice and promote a policy of exercise and physical activity referral for chronic musculoskeletal disease that utilises community engagement effectively and enacts change ‘together’.

## Supplementary Information

**Additional file 1.** Plain Language Summary.

**Additional file 2. **SPIRIT 2013 Checklist.

**Additional file 3.** GRIPP2 long form.

**Additional file 4.** Semi-structured interview guide for physiotherapists in pilot sites.

**Additional file 5. **Patient outcomes.

## Data Availability

Data sharing is not applicable to this protocol manuscript as no datasets were generated or analysed.

## Abbreviations

CFIR: Consolidated Framework for Implementation Research
ERIC: Expert Recommendations for Implementing Change
EQ-5D: 5-dimension EuroQol (EQ-5D)
GLA:D: Good Life with osteoArthritis from Denmark
GP: General Practitioner
ICER: Incremental cost-effectiveness ratio
IMPACT: Implementation of clinical guidelines for osteoarthritis together
OA: Osteoarthritis
QALY: Quality-adjusted life years
REC: Research ethics committee
RCT: Randomised controlled trials
SPIRIT: Standard Protocol Items: Recommendations for Interventional Trials
TILDA: The Irish Longitudinal Study on Ageing
