# Supplementary material for: Implementation of clinical guidelines for osteoarthritis together (IMPACT): protocol for a participatory health research approach to implementing high value care
Source: BMC Musculoskelet Disord. 2022 Jul 5;23:643. doi: 10.1186/s12891-022-05599-w (PMC9254615; doi:10.1186/s12891-022-05599-w)
Supplement: Supplementary file 1 — Additional file 1. Plain Language Summary. [file 12891_2022_5599_MOESM1_ESM.docx]

**Plain Language Summary**

**Title:** Implementation of clinical guidelines for osteoarthritis together (IMPACT): protocol for a participatory health research approach to implementing high value care.

**Summary:** Osteoarthritis is a disease of the joints, affecting 1 in 8 Irish people as they get older. Due to the growth of our ageing population amongst other reasons, this figure is expected to double within one generation. Most people with the disease in their hips or knees are likely to experience some level of pain as well as difficulty getting around and carrying out work or activities of daily living. Replacing the joint is a common treatment strategy and is normally a final resort that is given to few. Despite this, waiting lists for orthopaedic surgery are at their highest ever and patients are not offered many evidence-based management options in the meantime. Today, exercise and education programmes, such as the GLA:D^®^ initiative (Good Life with osteoArthritis Denmark), are recommended worldwide as an effective treatment for painful joints in middle-aged and older people. However, recommendation is one achievement. Actually putting this recommendation into practice has proved difficult. Therefore, this project aims to explore how to make sure that exercise and education programmes for patients with hip and knee problems are an available option for all patients, regardless of age, severity of disease, location and budget. To achieve this aim, a group of expert researchers, patients, patient advocacy groups, health professionals and decision makers will work together to decide on the best strategies to not only get this programme off the ground within the health service and community, but make sure it continues to be available once the research is over. The IMPACT project will also aim to see how this programme will change levels of pain, symptoms and quality of life and get people back to work for longer with less need for medication. This research has the potential to change the landscape and health service priorities for the management of osteoarthritis, by giving the power back to the patients and teaching them how to self-manage their pain through exercise and physical activity. This will only be achieved by bringing all parties to the table (patients, clinicians, researchers, decision makers), to bring about change ‘together’.
