# Supplementary material for: Implementation of clinical guidelines for osteoarthritis together (IMPACT): protocol for a participatory health research approach to implementing high value care
Source: BMC Musculoskelet Disord. 2022 Jul 5;23:643. doi: 10.1186/s12891-022-05599-w (PMC9254615; doi:10.1186/s12891-022-05599-w)
Supplement: Supplementary file 5 — Additional file 5. Patient outcomes. [file 12891_2022_5599_MOESM5_ESM.docx]

| ***Outcome Measures in GLA:D Ireland Registry*** | | | | |
| --- | --- | --- | --- | --- |
| ***Survey Section*** | ***Question*** | ***Answer options*** | ***Time*** | ***Reported by*** |
| *Patient Characteristics* | Sex | Female / Male / Other (specify) / Prefer not to say | Baseline | Patient |
| *Patient Characteristics* | Age | Age (years) | Baseline | Patient |
| *Patient Characteristics* | Marital status / Living situation | Single / Married or civil partnership / Co-habiting with partner / Separated / Divorced / Widowed | Baseline | Patient |
| *Patient Characteristics* | Educational Level (highest achieved) | Did not complete primary school / Completed primary school / Completed Junior Certificate / Completed Leaving Certificate or Secondary/High School equivalent / Completed apprenticeship / Completed Diploma / Completed undergraduate degree / Completed postgraduate degree | Baseline | Patient |
| *Patient Characteristics* | *(This question to be combined with healthcare utilisation questionnaire at baseline and 12 months)*  Do you have any of the following medical or physical conditions? (select all that apply)  No, I have no other medical or physical conditions / High blood pressure (hypertension) / High cholesterol / Rheumatological diseases (e.g. rheumatoid arthritis, fibromyalgia or lupus) NOT osteoarthritis? / Osteoporosis (thinning of the bones) / Back pain / Osteoarthritis in hand/finger joints / Chronic heart failure / Ischaemic heart disease (such as angina (chest pain from heart problem), heart attack, bypass surgery or angioplasty) / Other heart disease / Poor blood circulation in the legs (e.g. arteriosclerosis in the legs) / Anaemia (reduced level of red blood cells) or other blood disease / Stroke (incl. consequences after stroke) / Parkinson disease / Dementia / Other neurological disease (e.g. Multiple sclerosis, epilepsy or migraine) / Diabetes type 1 (no production of insulin) / Diabetes type 2 (low ability to use insulin or insufficient production of insulin) / Metabolic disorder (Hypo– or hyperthyroidism) / Kidney diseases / Ulcer or other stomach disease (e.g. gastritis or acid reflux) / Diseases in intestines (e.g. irritable bowel syndrome or chronic colitis/Chron’s disease) / Liver diseases / Chronic Obstructive Pulmonary Disease (COPD) / Asthma / Other chronic lung disease / Cancer – within last 5 years (except less severe cases of skin cancer) / Depression / Anxiety / Psoriasis / Obesity / Chronic fatigue syndrome, myalgic encephalitis (ME) / Other chronic diseases (diseases of long duration)___________ | Yes / No  If yes, Do you receive treatment for the condition? (Yes/No)  Does the condition limit your daily activities? (Yes/No) | Baseline, 3m, 12m | Patient |
| *Patient Characteristics* | Do you currently smoke? | Yes / No | Baseline | Patient |
| *Joint-related Characteristics* | Most affected joint (select ONE) | Right knee / Left knee / Right hip / Left hip | Baseline | Patient |
| *Joint-related Characteristics* | Other affected joints (select all that apply) | Right knee / Left knee / Right hip / Left hip | Baseline, 3m, 12m | Patient |
| *Joint-related Characteristics* | Have you previously had an injury to your most affected knee or hip that caused you to consult a doctor? | Yes /No | Baseline, 3m, 12m | Patient |
| *Joint-related Characteristics* | Have you had surgery on any of your hips or knees? (Baseline – therapist reported)  Since you started GLA:D, have you had surgery on any of your hips or knees? (3m Q -patient)  Since completing the last survey, have you had surgery on any of your hips or knees? (12m Q - patient) | Yes / No   - Which joint(s) have you had surgery on?   Right knee / Left knee / Right hip / Left hip   - What sort of surgery have you had on [joint]?   Total joint replacement, arthroplasty / partial joint replacement / arthroscopy, scope, Clean-out / ACL reconstruction (for knee only) / Other (free text) | Baseline, 3m, 12m | Patient |
| *Joint-related Characteristics* | Are you on a waiting list for a joint replacement for your hip/knee joint (if you are booked for joint replacement surgery, please answer 'yes')  Are you on a waiting list for a surgical opinion for your hip/knee joint?  Would you like to have surgery for your hip/knee joint? | Yes / No | Baseline, 3m, 12m | Patient |
| *Joint-related Characteristics* | In the past week what score would you give your hip/knee average pain out of 100? | 0-100 VAS | Baseline, 3m, 12m | Patient |
| *Joint-related Characteristics* | How long have you had the symptoms in your most painful knee/hip? | Months or Years | Baseline | Patient |
| *Joint-related Characteristics* | Has the patient had previous x-ray of most affected joint?  Did x-ray show OA? | No / Yes, more than 6 months ago / Yes, within last 6 months / Do not know  Yes/No/Do not know | Baseline | Patient |
| *Joint-related Characteristics* | **OA Checklist**  - Do you experience stiffness in your hip/knee first thing in the morning or after sitting for a while?  - Do you experience clicking or grinding in your hip/knee?  - Do you have less movement in your hip/knee than in the past?  - Do you have family members who have been diagnosed with osteoarthritis? | Yes / No  Combined with other information on age (40+), sex (female), overweight (BMI 25+), previous joint injury (Y) to diagnose clinical OA | Baseline | Patient |
| *Falls* | - Over the last year, have you had a fall? (A fall is defined as an event resulting in you inadvertently coming to rest on the ground or another lower level, with or without loss of consciousness or injury).  -If yes, how many falls did you have?  - Did you see a doctor or other healthcare professional because of at least one of your fall(s)? | Yes / No  One / Two / Three or more  Yes / No | Baseline, 12m | Patient |
| *Function, physical activity and exercise* | Do you have problems walking due to your hip/knee problems? | Yes / No | Baseline, 3m, 12m | Patient |
| *Function, physical activity and exercise* | UCLA Activity Score: Check one box that best describes current activity level. | 1: Wholly Inactive, dependent on others, and can not leave residence 2: Mostly Inactive or restricted to minimum activities of daily living  3: Sometimes participates in mild activities, such as walking, limited housework and limited shopping 4: Regularly Participates in mild activities such as walking, limited housework and limited shopping 5: Sometimes participates in moderate activities such as swimming, bicycling, a long walk or could do unlimited housework or shopping 6: Regularly participates in moderate activities such as swimming, bicycling, a long walk or could do unlimited housework or shopping 7: Regularly participates in active events such as bicycling for a long time, golf or hard gymnastics/fitness 8: Regularly participates in active events, such as bicycling for a long time, golf or hard gymnastics/fitness 9: Sometimes participates in impact sports such as jogging, soccer, handball, badminton, tennis, skiing, heavy labor or backpacking 10: Regularly participates in impact sports such as jogging, soccer, handball, badminton, tennis, skiing, heavy labor or backpacking | Baseline, 3m, 12m | Patient |
| *Function, physical activity and exercise* | Are you afraid that your joints will be damaged from physical activity and exercise? | Yes / No | Baseline, 3m, 12m | Patient |
| *Pain killers and sick leave* | Do you take any pain medications including herbal or dietary supplements? (select all that apply) | No I do not take any medication for my joint / Paracetamol (e.g. Panadol) / Non-steroidal anti-inflammatory, NSAID (e.g. ibuprofen, diclofenac, Nurofen, Buplex, Advil, Brufen, Easofen, Voltaren) / Topical NSAID cream (e.g. Voltaren cream/gel, Phorpain gel, Ibuleve gel, Nurofen gel) / Glucosamine / Hyaluronic acid injection / Corticosteroid (cortisone) injection / Morphine or other opioids / Tramadol / Codeine (e.g. Solpadeine) / Antidepressants (for pain / Anticonvulsants (for pain) / Methotrexate / Biphosphonate (e.g. Fosamax, Actonel) / Herbal supplements / Cannabis / Cannabidiol (CBD) containing products / Other (free text) | Baseline, 3m, 12m | Patient |
| *Pain killers and sick leave* | **Healthcare utilisation questionnaire**  Please indicate if in the PAST 3 MONTHS, you have visited the following practitioners or received the following services (exclude visits/services as an inpatient):  Physician (emergency room doctor)  Physician (GP/family doctor)  Surgeon  Other consultant  Physiotherapist  Chiropractor  Massage Therapist  Magnetic Resonance Imaging (MRI)  Computer Tomography (CT scan)  Radiographs (x-rays)  Other (please specify)  In the past 3 months, have you been admitted to hospital for any reason?  Do you currently have (select all that apply):  Are you currently receiving treatment for any ongoing illnesses or injuries?  Do you take medications for any other reasons (not for pain) on a regular basis? (e.g. hypertension, asthma, antidepressants etc.)  Are you currently taking any supplements? (vitamins, minerals, protein powder etc.) | For each: Do not know (Y/N) Visited (Y/N), Number of visits for your hip or knee (Number), Number of visits for reasons other than your hip or knee (Number).  Yes / No  If Yes, please describe any hospitalisations you have had in the past year below (Primary reason, number of nights in hospital, did you have surgery, describe surgery).  Private insurance / Medical card / Neither  No / Yes / Don’t know / Refuse  If yes, please describe the treatments to the best of your ability.  No / Yes  If yes, please list:  No / Yes  If yes, please list: | Baseline, 3m, 12m | Patient |
| *Pain killers and sick leave* | Current employment (select all that apply) | Unemployed / Part-time employment (less than 30 hours per week) / Full-time employment / Home duties / Studying full-time / Studying part-time / Retired / On leave | Baseline, 3m, 12m | Patient |
| *Pain killers and sick leave* | Have you been on sick leave because of your knee/hip in the last year? | No / Yes - less than 1 month / Yes - 1-3 months / Yes - more than 3 months | Baseline, 3m, 12m | Patient |
| *Quality of life* | **EQ-5D-5L** - Under each heading, please tick the ONE box that best describes your health TODAY.  MOBILITY;  SELF-CARE;  USUAL ACTIVITIES (e.g. work, study, housework, family or leisure activities);  PAIN / DISCOMFORT;  ANXIETY / DEPRESSION | 1 I have no problems in walking about 2 I have slight problems in walking about 3 I have moderate problems in walking about 4 I have severe problems in walking about 5 I am unable to walk about  1 I have no problems washing or dressing myself 2 I have slight problems washing or dressing myself 3 I have moderate problems washing or dressing myself 4 I have severe problems washing or dressing myself 5 I am unable to wash or dress myself  1 I have no problems doing my usual activities 2 I have slight problems doing my usual activities 3 I have moderate problems doing my usual activities 4 I have severe problems doing my usual activities 5 I am unable to do my usual activities  1 I have no pain or discomfort 2 I have slight pain or discomfort 3 I have moderate pain or discomfort 4 I have severe pain or discomfort 5 I have extreme pain or discomfort  1 I am not anxious or depressed 2 I am slightly anxious or depressed 3 I am moderately anxious or depressed 4 I am severely anxious or depressed 5 I am extremely anxious or depressed | Baseline, 3m, 12m | Patient |
| *KOOS-12 (knee) or HOOS-12 (hip) Questionnaires* | **Knee Injury and Osteoarthritis Outcome (KOOS) Questionnaire**  INSTRUCTIONS: This survey asks for your views about your knee. Answer every question by marking the appropriate box, only one box for each question. If you are unsure about how to answer a question, please give the best answer you can. **Pain** 1. How often do you experience knee pain? What amount of knee pain have you experienced the last week during the following activities? 2. Walking on a flat surface 3. Going up or down stairs 4. Sitting or lying **Function, daily living** The following questions concern your physical function. By this we mean your ability to move around and to look after yourself. For each of the following activities please indicate the degree of difficulty you have experienced in the last week due to your knee. 5. Rising from sitting 6. Standing 7. Getting in/ot of a car 8. Twisting/pivoting on your injured knee **Quality of life** 9. How often are you aware of your knee problem? 10. Have you modified your life style to avoid potentially damaging activities to your knee? 11. How much are you troubled with lack of confidence in your knee? 12. In general, how much difficulty do you have with your knee?  **Hip Injury and Osteoarthritis Outcome (HOOS) Questionnaire:**  *INSTRUCTIONS: This survey asks for your views about your hip. Answer every question by marking the appropriate box, only one box for each question. If you are unsure about how to answer a question, please give the best answer you can.*  *Pain*  1. How often do you experience hip pain?  *What amount of hip pain have you experienced the last week during the following activities?*  2. Walking of a flat surface  3. Going up or down stairs  4. Sitting or lying  Function, daily living  *The following questions concern your physical function. By this we mean your ability to move around and to look after yourself. For each of the following activities please indicate the degree of difficulty you have experienced in the last week due to your hip.*  5. Rising from sitting  6. Standing  7. Getting in/out of a car  8. Walking on an uneven surface  *Quality of life*  9. How often are you aware of your hip problem?  10. Have you modified your life style to avoid potentially damaging activities to your hip?  11. How much are you troubled with lack of confidence in your hip?  12. In general, how much difficulty do you have with your hip? | **KOOS-12:**  1: Never/Monthly/Weekly/Daily/Always  2: None/Mild/Moderate/Severe/Extreme  3: None/Mild/Moderate/Severe/Extreme  4: None/Mild/Moderate/Severe/Extreme  5: None/Mild/Moderate/Severe/Extreme  6: None/Mild/Moderate/Severe/Extreme  7: None/Mild/Moderate/Severe/Extreme  8: None/Mild/Moderate/Severe/Extreme  9: Never/Monthly/Weekly/Daily/Constantly  10: Not al all/Mildly/Moderately/Severely/Totally  11: Not al all/Mildly/Moderately/Severely/Extremely  12: Not al all/Mildly/Moderately/Severely/Extreme  **HOOS-12:**  1: Never/Monthly/Weekly/Daily/Always  2: None/Mild/Moderate/Severe/Extreme  3: None/Mild/Moderate/Severe/Extreme  4: None/Mild/Moderate/Severe/Extreme  5: None/Mild/Moderate/Severe/Extreme  6: None/Mild/Moderate/Severe/Extreme  7: None/Mild/Moderate/Severe/Extreme  8: None/Mild/Moderate/Severe/Extreme  9: Never/Monthly/Weekly/Daily/Constantly  10: Not at all/Mildly/Moderately/Severely/Totally  11: Not at all/Mildly/Moderately/Severely/Extremely  12: Not al all/Mildly/Moderately/Severely/Extreme | Baseline, 3m, 12m | Patient |
| *Anxiety and Depression* | **Hospital Anxiety and Depression Scale (HADS)** *Tick the box beside the reply that is closest to how you have been feeling in the past week.*  *Don’t take too long over you replies: your immediate is best.*  I feel tense or 'wound up':  I still enjoy the things I used to enjoy:  I get a sort of frightened feeling as if something awful is about to  happen:  I can laugh and see the funny side of things:  Worrying thoughts go through my mind:  I feel cheerful:  I can sit at ease and feel relaxed:  I feel as if I am slowed down:  I get a sort of frightened feeling like 'butterflies' in the stomach:  I have lost interest in my appearance:  I feel restless as I have to be on the move:  I look forward with enjoyment to things:  I get sudden feelings of panic:  I can enjoy a good book or radio or TV program: | Most of the time / A lot of the time / From time to time, occasionally / Not at all  Definitely as much / Not quite as much / Only a little / Hardly at all  Very definitely and quite badly / Yes, but not too badly / A little, but it doesn’t worry me / Not at all  As much as I always could / Not quite so much now / Definitely not so much now / Not at all  A great deal of the time / A lot of the time / From time to time, but not too often / Only occasionally  Not at all / Not often / Sometimes / Most of the time  Definitely / Usually / Not often / Not at all  Nearly all the time / Very often / Sometimes / Not at all  Not at all / Occasionally / Quite often / Very often  Definitely / I don’t take as much care as I should / I may not take quite as much care / I take just as much care as ever  Very much indeed / Quite a lot / Not very much / Not at all  As much as I ever did / Rather less than I used to / Definitely less than I used to / Hardly at all  Very often indeed / Quite often / Not very often / Not at all  Often / Sometime / Not often / Very seldom | Baseline, 3m, 12m | Patient |
| *Global Perceived Effect* | In general, how is your hip/knee problem compared to before participating in the GLA:D program? | Much better, an important improvement / Better, enough to be an important improvement / Somewhat better, not enough to be an important improvement / About the same / Somewhat worse, not enough to be an important worsening / Worse, enough to be an important worsening / Much worse, an important worsening | 3m, 12m | Patient |
| *Satisfaction* | How satisfied are you with the GLA:D® programme? | Not at all satisfied / Not satisfied / Neutral (neither satisfied or dissatisfied) / Satisfied / Very satisfied | 3m, 12m | Patient |
| *Satisfaction* | How often do you use what you’ve learned in GLA:D®? | Never / Every month / Every week / Every day / Several times a day | 3m, 12m | Patient |
| *Functional test* | Time to complete the 40m walk test  Use of walking aid | Time in seconds  Yes / No. If yes, type______ | Baseline, 3m | Therapist  and patient |
| *Functional test* | 30 sec chair stand test – number of rises during test | Collected in whole number | Baseline, 3m | Therapist  and patient |
| *Functional test (optional test)* | Hop for distance test | Distance in cm | Baseline, 3m | Therapist  and patient |
| *Demographics* | Height | Measured in cm | Baseline | Therapist  and patient |
| *Demographics* | Body mass | Measured in kg | Baseline, 3m | Therapist  and patient |
| *Clinic Details* | Describe the type of clinic where the GLA:D programme was offered | Primary care / Public hospital / Private hospital / Private practice / Other, please describe | Baseline | Therapist |
| *Referral source* | How was the patient recruited/referred to the programme? | GP referral / Advanced Practice Physiotherapist Triage/Referral / Orthopaedic Consultant referral / Clinic waitlist / Other waitlist (please describe) / Patient self-referral / Other healthcare professional referral (please describe) | Baseline | Therapist |
| *Delivery* | How many exercise sessions did you complete in a FACE TO FACE GROUP?  How many exercise sessions did you complete in AN ONLINE GROUP? 0-12 or more  How many exercise sessions did you complete on your own at home? How many education sessions did you attend? | Exercise - 0-12 or more  Education - 0 / 1 / 2 or more | 3m | Therapist  and patient |
